# Effect of fibrinogen concentrate on clot strength in trauma: preliminary results of an *in vitro* study

**DOI:** 10.1186/1757-7241-20-S1-O2

**Published:** 2012-03-22

**Authors:** MAS Meyer, AM Sørensen, PI Johansson

**Affiliations:** 1 Section for Transfusion Medicine, Capital Region Blood Bank, Copenhagen University Hospital, Copenhagen, Denmark; 2 Department of Anaesthesia and the Trauma Center, Center of Head and Orthopaedics, Copenhagen University Hospital, Copenhagen, Denmark

## Background

Fibrinogen supplementation in trauma has been suggested in order to restore or improve haemostatic competence; this could possibly reduce or replace the need for transfusions in case of bleeding. Results from functional haemostatic assays indicate that lowered clot strength is associated with a risk for massive transfusions [1]. The optimal fibrinogen concentration or indications for supplementation in trauma patients have not been established [2].

## Aim

To examine the *in vitro* effect of adding fibrinogen concentrate to whole blood from trauma patients by Thrombelastography (TEG).

## Methods

Eleven patients with severe injury admitted to a Danish level 1 trauma centre were enrolled in the study. Inclusion was based on: systolic pressure < 100 mmHg and/or GCS ≤ 8 and/or substantial bleeding. Eight out of eleven patients received a transfusion within 12h after hospital admission. Mechanisms of injury included: road traffic accidents, fall injuries, and stab- and gunshot wounds. A citrated blood sample was obtained at admittance. TEG analyses were performed using both citrated kaolin (CK) and functional fibrinogen (FF). CK clot strength (maximum amplitude; MA) reflecting both the platelet and the fibrinogen contribution were compared to that of FF, which solely reflects fibrinogen contribution to clot strength. Volumes of fibrinogen concentrate equivalent to 6g*75kg^-1^ were added to samples prior to TEG analysis. *p* < 0.05 was considered significant.

## Results

Fibrinogen concentrate increased the clot strength in both CK and FF assays (Fig.1). CK MA increased by 8% (*p* = 0.013) and FF MA by 44% (*p* = 0.005) after addition of fibrinogen concentrate.

## Conclusions

In whole blood from trauma patients with severe injury, fibrinogen concentrate administered in a dose equivalent to 6g*75kg^-1^ increased clot strength significantly. These results indicate a possible pro-haemostatic effect of fibrinogen concentrate in severely injured trauma patients.
